# From structural modalities in perinatal medicine to the frequency of preterm birth

**DOI:** 10.1007/s00281-020-00805-0

**Published:** 2020-08-25

**Authors:** Philipp Deindl, Anke Diemert

**Affiliations:** 1 Department of Neonatology and Pediatric Intensive Care Medicine, University Children’s Hospital, University Medical Center Hamburg-Eppendorf, Hamburg, Germany; 2 Department of Obstetrics and Fetal Medicine, University Medical Center Hamburg-Eppendorf, Martinistr. 52, D-20246 Hamburg, Germany

**Keywords:** Preterm birth, Prematurity, Neonatal care, Prenatal care, Cervical insufficiency, Progesterone, Immunology of parturition, Risk factors prematurity, Genetics of prematurity

## Abstract

Preterm birth is a global health problem that affects 11% of all live births: it remains a significant cause of death and an important cause of long-term loss of human potential among survivors all around the world. In the last decade, many developed countries have seen an increase in the rate of infants born preterm. Various theoretical and practical concepts have been discussed that aim to optimize the perinatal care of preterm infants and their mothers. These include the definition of hospitals with different levels of care, the regionalization of care, the creation of large care units with high case numbers, and a high level of expertise. This review article focuses on the connection between health care structures and different aspects of preterm birth. Furthermore, this review article highlights the fact that preterm birth is a heterogeneous syndrome with many underlying pathologies and that the causality for a large number of cases remains unexplained. There is still a significant knowledge gap concerning the actual drivers of spontaneous preterm birth, and future research particularly in parturition immunology as well as genetics of prematurity will be essential to identify new targets for therapy.

## Introduction

Worldwide, about 11% of all children are born preterm, resulting in a total of 15 million children born before the 37th week of gestation. In many countries, the rate of preterm births is increasing, and the survival rate of preterm babies has dramatically improved in developed countries [1–3]. The survival of children born pretermly depends on the available resources in obstetrics and neonatal care as wells as on the attitudes towards viability in the individual countries [4]. Preterm birth represents a significant cause of death and can lead to serious harm to survivors all around the world [2]. Especially, the groups of extremely preterm (EPT) and extremely low birth weight (ELBW) babies require considerable resources and highly specialized treatment. Preterm birth has a severe impact on the morbidity and mortality of newborns. Medical complications can result in life-long limitations, which, in turn, places a heavy burden on the families concerned and involves high socio-economic costs [4, 5]. In about half of all neonatal deaths, prematurity is a potential risk factor [6]. Very preterm babies (< 32-week gestation) make up only a small percentage of live births (1.6%). This small group of children, however, accounts for more than half of all neonatal deaths, often suffers severe complications with long-term impairment, and thus causes considerable health care costs [4, 7, 8].

Various theoretical and practical concepts aim to optimize the perinatal care of preterm infants and their mothers. These include the definition of hospitals with different levels of care, the regionalization of care, the creation of large care units with high case numbers, and a high level of expertise. This review article focuses on the connection between health care structures and different aspects of preterm birth.

### Definitions

#### Preterm birth

According to the WHO definition, a child is born preterm if it is born before the completed 37th week of gestation or with fewer than 259 days since the first day of the mother’s last menstrual period. Depending on completed gestational age in weeks, preterm neonates are divided into extremely preterm (< 28), very preterm (28–31), and moderately preterm born neonates (≥ 32). From the 34th week of gestation onwards, these children are also referred to as late preterm neonates [2]. Even children born at 37 or 38 weeks gestational age have a detectably inferior average outcome than babies born at 40 weeks gestational age [9].

#### Stillbirth

A stillbirth is defined as a child born with a weight of more than 1000 g or with a gestational age of 28 weeks [10]. However, the official definition of stillbirth is subject to change in many countries. Often also, the medical team’s opinion of the child’s chances of survival plays a role in the classification of a stillbirth. Since about 80% of stillbirths in high-income countries are born preterm, the actual burden of preterm birth is likely to be underestimated when analyzing live births alone [10].

### Epidemiology of preterm birth

In many countries, the rate of children born too early is increasing and varies between 10 and 15%, depending on the region and country [2]. Preterm birth is the most critical single risk factor for perinatal and neonatal mortality and represents, therefore, a tremendous global challenge for health systems [11]. The causes of the increase in preterm births worldwide are still unclear. To analyze the risk factors for both spontaneous preterm birth and provider-associated preterm birth, Ferrero et al. examined data from over four million single births in four countries with high average income. The study failed to identify specific factors, which makes the possibilities for intervention by political, structural, organizational changes seem very limited. The authors conclude that the focus of research should, therefore, shift to the biological causes of preterm birth [11].

Over three-quarters of all preterm babies are born between the 32nd and 36th week of gestation. Most of these moderate preterm babies survive with little supportive therapy. Nevertheless, these children have a higher relative risk of dying within the first year of life than term infants. Due to their sheer number, these children account for an essential proportion of infant mortality [12]. The short-term morbidity of preterm babies between the 34th and 36th week of pregnancy (intraventricular bleeding and respiratory problems) is also significantly higher compared to term infants. Besides, there are long-term sequelae such as possible developmental neurological impairment, poorer performance at school, and a higher risk of cerebral palsy. Therefore, this patient group is of the most considerable importance in service planning, primary neonatal care, and specialized care for moderately preterm infants [7, 8, 13].

### Preterm birth—a heterogeneous syndrome with multiple underlying pathologies

Preterm birth has various causes, but there are two major categories: spontaneous preterm birth due to spontaneous onset of labor or following prelabor preterm rupture of membranes (pPROM), and the provider-initiated preterm birth, by induction of birth or elective cesarean section before the completed 37th week of pregnancy. In the case of provider-initiated preterm birth, a distinction is made between infant, maternal, and other non-medical indications [14].

In up to half of the cases of preterm birth, the cause remains unexplained. Social and environmental factors are known to influence the risk of preterm birth. Maternal risk factors include a low BMI, very young or advanced age, and short intervals between consecutive pregnancies. The availability of assisted conception resulted in an increased rate of multiple pregnancies, which increase the risk of preterm birth tenfold due to uterine overdistension.

Boys are more frequently born prematurely (55% of all preterm births), with males of similar gestation having a poorer prognosis than girls [15]. Genetic factors modulate the length of regular gestational length, neonatal respiratory distress, and neonatal mortality [16]. The reasons for preterm birth are complex and often occur in combination. Individual behavioral and psychosocial factors, environmental influences, infertility treatments, and biological and genetic factors are likely to influence the risk of preterm birth.

### Health consequences of preterm birth

The chances to survive as an extremely preterm infant vary depending on medical resources available in obstetrics and neonatal care [4]. In developed countries, the survival of these patients has dramatically improved. In some countries, the critical limit of viability has, therefore, been extended to 22 weeks gestation. In developing countries, however, these children only survive very rarely. The improved survival of these high-risk patients is due to advances in obstetrics and antenatal care, as well as improved structures and strategies for postnatal resuscitation and stabilization. Immaturity in the context of preterm birth affects all organ systems with severe and extensive consequences for the physical, neurodevelopmental, and behavioral development of these children [2]. Physical impairments in preterm babies can involve the lungs in the form of chronic lung disease of prematurity, myopia, hearing loss, and cardiovascular problems. Neurological sequelae can include global developmental delay, executive functioning, and behavioral problems [9, 17]. Adams-Chapman et al. evaluated the neurodevelopment of 2113 extremely preterm infants and concluded that 59% of the examined children presented with normal findings between 18 and 26 months’ adjusted age, respectively, while 19% presented slightly abnormal and 22% definitively abnormal. A shift towards less severe motor and sensory neurodevelopmental impairment was observed over time in their sample of patients [18].

### Economic consequences of preterm birth

In 2005, the Institute of Medicine (IOM) estimated that the yearly costs of preterm birth in the US, including the costs of delivery, medical care up to the age of five, but also the lifelong costs of specific neurodevelopmental impairment, and the resulting reduced productivity amounted to about $26 billion. Additional long-term conditions threaten preterm babies in their further development: asthma, hypertension, insulin resistance, learning, and behavioral problems [5]. The IOM report and other studies identified the highest total costs for medical care among extremely preterm infants < 28 weeks of gestation [19]. The health care costs of prematurity depend, on the one hand, on the gestational age, but also the relative number of patients. The majority of preterm infants fall into the category of late-preterms and, therefore, account for a relevant share of the overall health care costs.

### The burden of prematurity for families

Families with a preterm baby born are faced with numerous burdens, especially if the child has severe impairments. Costs are incurred for child care of siblings during hospitalization and outpatient appointments, transport costs, and additional expenses for accommodation. Family members usually care for these patients, which may affect their employment situation and often means financial losses in family income [20]. The burden on a family with a preterm child goes far beyond the purely financial costs. Long-term disabilities of the new family member can tremendously affect the family life of parents, siblings, and extended family members. The strains on family members depend on both the physical health condition and the neurological outcome of the preterm infant and can lead to depression, parental, and family conflicts [4, 5].

**Fig. 1:**
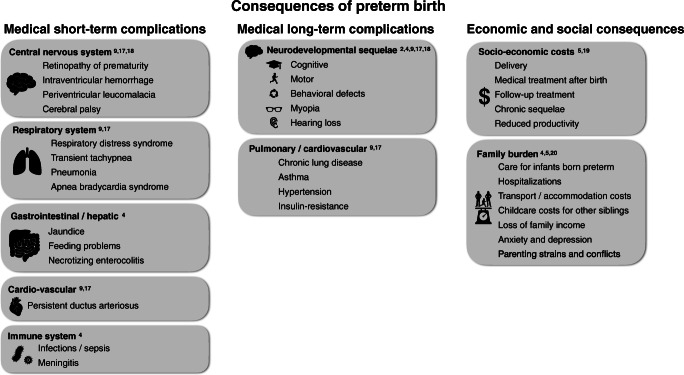
Consequences of preterm birth stratified by medical short-term, medical long-term, and economic and social consequences

Figure 1 summarizes risk factors and health consequences of preterm birth.

### How vital is the level of care at birth for high-risk preterm infant survival?

Numerous studies have shown that hospital structures have a considerable influence on the outcome of preterm infants. An extensive meta-analysis by Lasswell et al. summarized studies over 30 years to investigate the relationship between hospital level at birth and neonatal mortality for high-risk infants. The authors reported that the birth of a very low birth weight (VLBW) infant and VPT infant outside a level III hospital represented a significant risk factor for higher neonatal mortality—an observation that was evident over the entire observation period of 30 years [21]. When limiting the analysis to only high-quality studies with a total of over 45,000 children included, the risk of death was estimated to be 60% higher for VLBW children and even 80% higher for ELBW children born at a non-level III hospital compared to those born at a level III hospital [21].

### Hospital characteristics as performance indicators

Based on the data of the Vermont-Oxford network on VLBW infants between 2010 and 2013 treated in 862 NICUs, Rochow et al. simulated how two different strategies for forming larger NICUs would affect neonatal mortality [22]. Step by step, individual NICUs were excluded from the model simulation based on the one hand on the NICU admission volume and, on the other hand, on NICU quality cut-offs. The reduction in NICUs based on quality criteria more effectively improved system mortality compared to a reduction based on the admission volume. The mortality rate was improved by 5% after reducing 8% of NICUs and redirecting 6% of infants. The authors concluded that a minimum number of admissions may be necessary to maintain the skills of the treatment team, but that quality criteria for evaluating hospitals should also be taken into account when restructuring neonatal care [22]. By analyzing data from all hospitals in California between 1991 and 2000 on mortality of VLBW infants, Phibbs et al. found that VLBW mortality was lowest over the entire period among children born in hospitals with both high VLBW admission volume and a high level of care. As a consequence, they postulated that an increased regionalization towards high-level NICUs might reduce mortality among very-low-birth-weight infants [23]. Jacob et al. analyzed the causes of death in 641 infants in order to identify potentially preventable factors that could improve neonatal care. The causes of death were manifold and strongly dependent on the gestational age. In children born prematurely, causes of death directly related to preterm birth predominated. Birth at a center without adequate care facilities was identified as a potentially modifiable risk factor for death. Preterm infants treated by specialized maternal-fetal teams and receiving prenatal steroids survived more often [24].

From an organizational and logistical point of view, there are many arguments in favor of centralizing the highly specialized care of preterm infants. Centralization allows the concentration of cost-intensive technologies and the development of highly specialized expertise among nursing staff and physicians in a few locations.

### Prevention of preterm birth

#### Identification of risk factors for preterm birth in prenatal care

A comprehensive assessment of risk factors for preterm birth early in pregnancy will be critically important for the prevention of preterm birth. Particular emphasis should be placed on risk factors for preterm birth that can be managed, such as smoking during pregnancy, adherence to dietary recommendations during pregnancy, and maternal stress. When taking the medical history, it is essential to consider that an interval between two pregnancies of less than 12 months is associated with a four-fold increase in risk for preterm birth. Another strong risk factor is a previous spontaneous preterm birth associated with an odds ratio for a repetition of 3.6 [25].

It is critically important to assess cervical length by ultrasound between gestational weeks 16 and 24. Previous studies have demonstrated that singleton pregnancies with cervical shortening to less than 25 mm without a history of preterm birth have a risk of 25–30% for prematurity [26]. The prematurity risk will further increase to more than 35% in women with cervical shortening plus a positive history for preterm birth [27] with the highest risk of more than 50% observed if the cervical length is less than 15 mm on ultrasound.

However, it is crucial to note that a substantial portion of pregnant women that will be affected by preterm birth does not have any discernible risk factors [11]. This finding undermines the hypothesis that preterm birth is a multifactorial syndrome and that many of the underlying environmental, genetic, and epigenetic factors are so far only poorly understood.

### Prenatal interventions to prevent preterm birth

Numerous studies investigated a variety of prenatal interventions to prevent preterm birth. The majority of interventional studies have focused on vaginally applied progesterone formulations to inhibit inflammation and cervical ripening and on mechanical techniques to address cervical insufficiencies such as cervical pessaries or invasive cervical cerclage.

A recent meta-analysis of randomized clinical trials that investigated progesterone treatment in singleton pregnancies with cervical shortening below 25 mm has established a significant benefit of vaginally applied progesterone with a 22.5% reduction of preterm births in the progesterone group compared to 14.1% in the placebo group. The progesterone treatment group had a relative risk of 0.62 (confidence interval 0.47–0.81) and showed improved neonatal outcomes (Romero R, 2018). Vaginal progesterone is the only intervention for the prevention of preterm birth that is recommended by guidelines in most countries [28].

Mechanical interventions to address cervical insufficiency have been widely used since the 1970s. However, while numerous trials have investigated the efficiency of these measures to prevent preterm birth, the evidence for cervical cerclage and cervical pessaries remains inconclusive. The lack of clarity is mainly because the majority of studies were performed over 10 years ago in very heterogeneous patient populations, and many of these are not meeting modern standards for follow-up and outcome reporting. Since large-scale meta-analyses of individual data from published studies on cervical pessaries and cervical cerclage have yielded contradictory results [29]), most authors recommend that these measures should only serve as a bail-out approach in selected cases.

Epidemiological studies have identified several risk factors associated with preterm birth, such as genitourinary infections [30], nutritional deficiency, or active and passive smoking [31]. Consequentially, some intervention trials have investigated the effectiveness of anti-infective treatment, nutritional supplementation, and smoking cessation in the prevention of preterm birth. However, despite the strong risk-factor association observed in epidemiological studies, risk factor intervention studies for antibiotic treatment of bacterial vaginosis [32] or asymptomatic bacteriuria [33] did not have a discernible benefit on the rate preterm birth. Similarly, studies investigating nutritional supplementation with calcium, iron with or without folic acid, folic acid alone, vitamins A, D, E, and multivitamin preparations did not result in a significant reduction of prematurity in a recent meta-analysis of multiple randomized trials [34].

Smoking constitutes a common and modifiable risk factor for preterm birth. Encouragingly, national tobacco control programs for the prevention of passive smoking were associated with a reduction of prematurity in some European countries [35]. Furthermore, a Canadian smoking cessation study working with nicotine patch supplementation during pregnancy was associated with a reduced rate of preterm births [36].

**Fig. 2:**
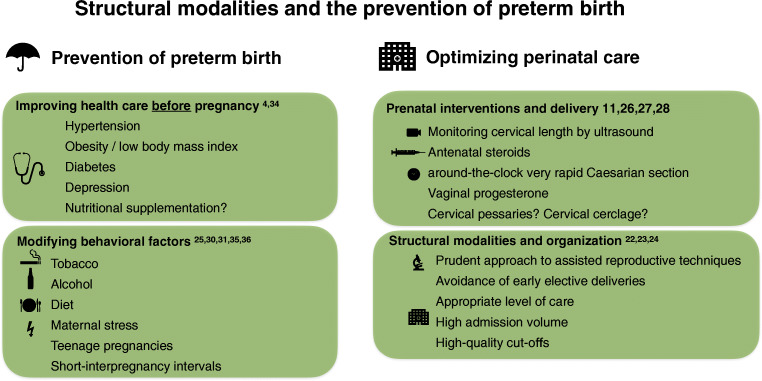
Structural modalities and the prevention of preterm birth—this figure summarizes strategies and recommendations for the prevention of preterm birth

A summary of recommendations and strategies for the prevention of preterm birth is displayed in Fig. 2.

## Conclusion and future directions of research

Prematurity represents a global health problem with an estimated 1.1 million neonatal deaths linked to complications of premature birth. Indeed, premature death is second only to pneumonia as the most common cause of mortality in children under the age of five. In many low- and middle-income countries, preterm birth is mostly a structural problem that must be addressed by socio-economic improvements, availability of prenatal care, and prevention of transmitted diseases. In contrast, in some high-income countries, the rate of preterm birth has increased in recent years due to the widening use of assisted reproduction techniques or medically induced induction of birth.

However, even when accounting for these medical practices and when comparing the best performing developed countries, there is still an increased rate of preterm birth in many high-income countries in Europe and North America in the last decade [37]. This worldwide “epidemic” of preterm birth affects low-, middle-, and high-income countries alike, and the gap is primarily a knowledge gap. So far, the available treatments and interventions for preterm birth have shown only limited success, and even in the best-performing countries, the residual rate of prematurity remains stubbornly high. Even in high-income countries with highly developed healthcare systems and prenatal care, two-thirds of preterm birth cases are not associated with known risk factors [11]. The underlying cause for the lack of success with most intervention trials for preterm birth so far is most likely our poor understanding of the underlying drivers for this disease. In order to develop targeted preventive strategies, we must come to a better understanding of the pathophysiology and develop a classification of preterm birth based on the underlying etiology.

It is well known that preterm birth displays significant genetic heritability, but very little is known about specific genetic factors that predispose to prematurity. Furthermore, a substantial amount of current research data indicates that many cases of preterm birth are based on a disorder of the feto-maternal immune adaptation [38]. Both on-time delivery and preterm birth are associated with the upregulation of inflammatory cells [39]. Apart from the particular case of intrauterine infections, it is still unclear how early activation of the inflammatory cascade, which is the triggering event of preterm birth, is activated in preterm birth.

Previous research approaches to clarify this question were primarily the targeted elimination of individual inflammation cascades by transgenic mouse models. It became clear, however, that the mechanisms were redundant since switching off individual genes or pathways is usually not sufficient to switch off maternal immune tolerance for the fetus [38].

In future research on the pathophysiology of preterm birth, it is, therefore, crucial to take a hypothesis-free approach. This means a departure from the analysis of individual inflammation genes or pathways towards a systems biological approach with the help of next-generation sequencing combined with proteome analyses on patient samples or controls. This approach provides an example of how basic research on preterm-birth might lead to better therapeutic approaches in the future.

## Data Availability

Not applicable.

## Abbreviations

VLBW: Very low birth weight
ELBW: Extremely low birth weight
VPT: Very preterm
NICU: Neonatal intensive care unit
PTB: Preterm birth
